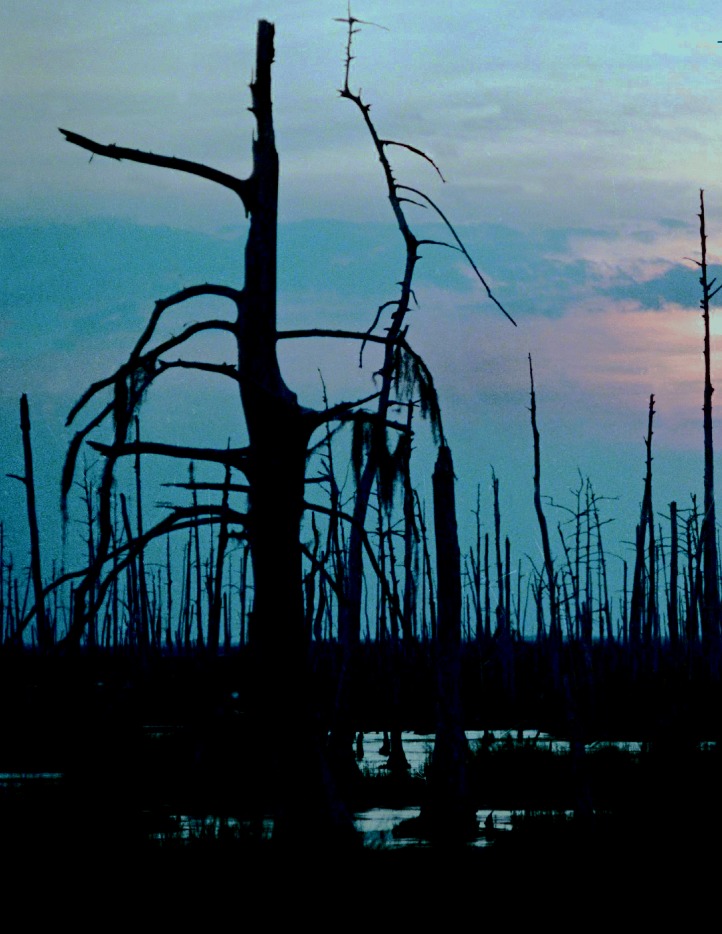# Louisiana’s Wetlands: A Lesson in Nature Appreciation

**DOI:** 10.1289/ehp.114-a40

**Published:** 2006-01

**Authors:** John Tibbetts

Hurricane Katrina’s disastrous flooding of the Gulf Coast confirmed three decades of warnings by scientists. Most of New Orleans is below sea level, and South Louisiana’s coastal wetlands, which once helped buffer the city from giant storms, have been disappearing at a spectacularly swift pace. Now some researchers are calling for restoration of wetlands and barrier islands to help protect New Orleans the next time a hurricane strikes.

An average of 34 square miles of South Louisiana land, mostly marsh, has disappeared each year for the past five decades, according to the U.S. Geological Survey (USGS). As much as 80% of the nation’s coastal wetland loss in this time occurred in Louisiana. From 1932 to 2000, the state lost 1,900 square miles of land to the Gulf of Mexico.

By 2050, if nothing is done to stop this process, the state could lose another 700 square miles, and one-third of 1930s coastal Louisiana will have vanished. Importantly, New Orleans and surrounding areas will become ever more vulnerable to future storms. “New Orleans can’t be restored unless we also address coastal and wetland restoration too,” says Craig E. Colten, a geographer at Louisiana State University (LSU).

## A River and a City

The vast watershed of the Mississippi River ranges from Montana in the west to New York state in the east. Spring rains send sediment-rich runoff into the river and its tributaries. For thousands of years, the Big Muddy has flowed down to the Gulf of Mexico, where great floods periodically burst over the riverbanks, allowing huge quantities of silt to settle and nourish wetlands. The land naturally sinks, or subsides, as loose sediments from the Mississippi River settle and compact.

The river slows as it reaches the gulf because of the tides pushing upstream; as it slows down, it spreads out and delivers much of its sediment load into deltaic deposits. The Mississippi Delta was fed by these influxes of mud, creating 5 million acres of South Louisiana before the twentieth century. Every millennium or so, the Mississippi River would change direction at its gulf outlet, meandering from east to west and back again. As a result, the river created six different delta “lobes” on which the entire coastline of South Louisiana was formed.

In 1718, French settlers founded New Orleans on a natural ridge of high land on a bend of the Mississippi River, with Lake Pontchartrain (which is actually an inlet of the Gulf of Mexico) to the north and coastal wetlands to the east, west, and south. But flooding was a problem. By 1812, the settlers had built levees on the east bank to Baton Rouge, 130 miles upstream, and on the west bank as far as Pointe Coupée, 165 miles upstream.

Over the next two centuries, the city drained surrounding wetlands to prevent disease and encourage development. The city eliminated swamps following mosquito-borne yellow fever epidemics that killed 40,000 residents between 1817 and 1905. As the city grew, the only lands available for development were low-lying areas north toward Lake Pontchartrain. At the turn of the twentieth century, the city created an integrated public works department, which was responsible for draining the wetlands.

“It was the draining of the lower areas that allowed suburbanization to occur,” says Colten. But the lowlands, originally just inches above sea level, steadily sank. “When you drain these areas, you suck the water out of the peaty soils, which begin to compress, or subside,” he says. “That’s why these areas have continued to subside.”

New Orleans also continually built higher and stronger levees to contain river flooding. In 1928, Congress authorized major levee improvements, and the U.S. Army Corps of Engineers began shoring up the flood control system, including levees, along the entire lower Mississippi and in New Orleans. By the 1950s, LSU geology professor James P. Morgan had begun to document dramatic rates of land loss in Louisiana’s coastal zone, which stretches 300 miles from the Texas border to the Mississippi state line and 50 miles inland.

## The River Today

Today, South Louisiana is one of most intensively engineered places in the nation. Vast quantities of water are diverted or rerouted through a lacework of navigation corridors held in place by 2,000 miles of earthen, rock, and concrete levees. Walled off from the floodplains, the river can no longer provide enough silt to the delta to keep up with natural subsidence and sea level rise. About two-dozen dams also hold sediment back from the river and its tributaries. “We have tamed the river for the almost exclusive benefit of navigation,” says David R. Conrad, a senior water resources specialist with the National Wildlife Federation.

The construction of high levees did end the spring floods along the lower Mississippi, but at an environmental cost, eventually eliminating many of the wetlands, floodplains, and barrier islands of the delta. “When you lose wetlands and flood-plains, you lose their natural services including storage capacity during floods, and when you lose coastal wetlands, you lose wave and storm protections,” says Sandra Postel, director of the Global Water Policy Project, a nonprofit organization based in Amherst, Massachusetts. “Katrina in South Louisiana was an example of what happens when you disturb the natural infrastructure.”

In November 2005, the National Academies released a report, *Drawing Louisiana’s New Map: Addressing Land Loss in Coastal Louisiana*. The report notes that building and maintaining levees and dams along the Mississippi River was a “more or less ubiquitous” cause of wetland loss. Another geographically widespread cause was voracious grazing by nutria, a nonnative species, which destroyed wetland vegetation.

But the report also points out that there were other causes “superimposed on these broad influences,” particularly including activities by the oil and gas industry. Peaking during the 1960s through the 1980s, oil and gas companies dredged canals for exploration. There are currently 10 major navigation canals and 9,300 miles of pipelines in coastal Louisiana serving about 50,000 oil and gas production facilities. These canals, which are perpendicular to the coast, have created new open water areas, drowning wetlands and allowing salt-water intrusion into freshwater ecosystems. The result—land loss hot spots. “There is also evidence,” the report says, “that extraction of large volumes of oil and gas has exacerbated the problems of inundation and saltwater intrusion”—that is, withdrawing oil and gas along geologic faults seems to exacerbate subsidence in coastal Louisiana.

The Mississippi Delta is also home to South Louisiana’s port complex, which lines both banks of the Mississippi River for 172 miles as well as points offshore, including the Port of New Orleans, the Port of South Louisiana, the Port of Baton Rouge, and the Louisiana Offshore Oil Port in the Gulf. Because of its size and location, adjacent to oil and gas refineries and drilling platforms, this port complex is one the most important in the United States. Louisiana’s coastline produces one-fifth of the country’s oil and one-quarter of its natural gas. Through South Louisiana’s ports the bulk commodities of U.S. agriculture—corn, wheat, and soybeans—are sent around the world, and the bulk commodities needed for American industry—steel and concrete, for instance—come into the country.

The Mississippi River Gulf Outlet, a little-used 40-year-old shipping channel connecting the Gulf of Mexico to the Mississippi River, is believed to have served as a funnel for Katrina’s storm surge. The navigation channel and the eastern levee of the Mississippi River seem to have directed high water into the Breton Sound estuary southeast of New Orleans, according to Greg Steyer, a USGS wetland scientist. From there, the surge poured into Lake Pontchartrain and an industrial canal, where it overwhelmed levees, contributing to flooding in St. Bernard Parish and the Lower Ninth Ward of New Orleans. Like the oil and gas canals, the outlet also allows saltwater intrusion and tidal action into freshwater ecosystems, killing vegetation and turning the marsh into a stretch of open muddy water.

The Gulf of Mexico is also subject to the general sea level rise being observed worldwide, with potential ramifications for the Gulf Coast. Over the past century, the warming climate has pushed up mean sea level four to eight inches worldwide, and computer models suggest that this rise will probably accelerate, according to a 2001 report of the U.S. Global Change Research Program, *Climate Change Impacts on the United States: The Potential Consequences of Climate Variability and Change*. By 2100, global sea level is projected to rise an additional 19 inches along most of the U.S. coastline.

## Death of the Wetlands

This combination of factors has killed wetlands in South Louisiana from the inside out. “Some of the inner marshes have actually eroded faster than some of the extreme coastal areas,” says Gary Fine, manager of the Natural Resources Conservation Service’s Golden Meadow Plant Materials Center in Galliano, Louisiana. In the delta, sediment deposits from tidal creeks and rivers build up the banks, creating modest natural ridges. Land elevations fall toward the center of coastal marshes, freshwater swamps, and bald cypress forests. Starved of new sediments and flooded by tides, the inner areas become constantly submerged. “Especially in the salt marshes,” Fine explains, “the plants start dying in the center due to rising water and decreasing sediments, and then the loss expands outward to the edges.” As a result, South Louisiana has become a patchwork of open water and remnant wetlands.

“By 2050, the city will be closer to and more exposed to the Gulf of Mexico,” noted authors of a restoration proposal, *Coast 2050: Toward a Sustainable Coastal Louisiana*. Hurricane Katrina itself pushed the city closer to the coast. The hurricane, making landfall in lower Plaquemines Parish, had a storm surge of almost 30 feet, which caused extensive erosion at the coastal edge. For example, Katrina almost wiped out the Chandeleur Islands, a 40-mile-long series of uninhabited barrier islands southeast of New Orleans. “The sand and marsh are gone,” says Asbury Sallenger, an oceanographer with the USGS Center for Coastal and Watershed Studies in St. Petersburg, Florida. “Before Katrina, the islands were five meters high; now there’s a less than half a meter left.”

Gregory W. Stone, a coastal geologist at LSU, says that if the current trend of wetland loss and barrier island erosion continues, it will worsen the effects of future hurricane surges in South Louisiana. “Storm surge and storm waves will increase if we lose more wetlands and our barrier coast,” he says. “Wetlands and barrier islands are the first line of defense. That means areas such as New Orleans would become more vulnerable to inundation.”

Further land loss would also endanger oil and gas facilities, the huge port complex, and the gulf’s valuable fishing industry. South Louisiana’s wetlands are critical nursery areas for commercially important marine species, including shrimp, blue crabs, oysters, redfish, and menhaden. Land loss in South Louisiana, says Stone, “is not a local problem—it’s a national problem.”

## Restoration Plans

In an effort to rebuild the state’s natural infrastructure, Congress passed the 1990 Coastal Wetlands Planning, Protection, and Restoration Act, sponsored by Senator John Breaux (D–LA). The Breaux Act provides about $50 million each year for wetlands restoration projects in Louisiana. The Breaux Act has provided funding for 118 restoration projects, and 75 projects have already been built. But most of these projects are relatively small in scale.

In 1996, the state of Louisiana and a group of federal agencies joined with parish officials and the public to create a consensus document. The result, after 65 public meetings over 18 months, was *Coast 2050*, which outlined strategies and measures needed to restore the state’s wetlands and barrier islands.

*Coast 2050* proposed that the Mississippi River be re-engineered to imitate natural processes. That is, some portion of the river’s flow should be re-diverted via pipelines or canals to flush into the delta so that South Louisiana’s sinking ecosystems could be built up. “*Coast 2050* essentially calls for putting holes in the straitjacketed Mississippi River,” says Conrad. “This process could be one of the most interesting and expensive and important environmental engineering processes ever. It is a huge opportunity to put things back together if we have the will.”

These water diversions would feed freshwater marshes and control saltwater intrusion from being pushed upriver by the rising sea level. The Caernarvon Freshwater Diversion Project, funded in the mid-1980s, could be one model for this approach. The diversion consists of a $26-million opening in the river levee built by the Army Corps about 24 miles south of New Orleans. A concrete culvert diverts water into a canal that feeds marshes behind Breton Sound, which had been losing land. This diversion has been shown to increase marsh and freshwater plant acreage.

*Coast 2050* also recommended that federal agencies dredge soils and ancient sand-bars to create new marshlands; plug up the Mississippi River Gulf Outlet; and shore up barrier islands that are the first line of defense against approaching hurricanes. However, the cost cited in the report for all these projects seemed too huge to consider: $14 billion (by comparison, estimates for rebuilding after the 2005 hurricane season have been placed as high as $200 billion).

Kerry St. Pé, director of the Barataria-Terrebonne National Estuary Program, says there’s no time to waste. Freshwater diversions alone are not enough to solve the land loss problem, he adds. Dredge material should be pumped immediately via pipes from navigation channels in the delta, including the Mississippi River, to shore up hot spots of wetland loss. “We need the sediment now,” he says. The Corps of Engineers already dredges 40–45 million cubic yards of sediment from the delta’s numerous navigation channels each year, he says, and the material is discharged off the end of the continental shelf because that’s the least expensive method of disposal. “We could use that sediment to build wetlands,” says St. Pé.

From 2000 through 2003, the Corps of Engineers and the state of Louisiana collaborated on a feasibility study for a $17-billion coastal restoration plan lasting 30 years. Yet this study, based on *Coast 2050*, also seemed far too expensive at the time. “It never went up to Congress because it exceeded what potentially could be funded,” says Steyer. “We were asked to focus it on more of the near term, over ten years, addressing what are the critical projects that could be done.”

In November 2004, state and federal agencies proposed a near-term effort, the Louisiana Coastal Area Ecosystem Restoration Study. The findings from this study led to the 2005 Water Resources Development Act, which calls for Congress to spend $1.9 billion over 10 years on restoration efforts in the delta; the bill is still being worked out in Congress. The act—intended to be a first, smaller step toward a 30-year $17-billion plan—follows the strategies of *Coast 2050*, says Steyer.

However, Oliver Houck, who directs the environment program at Tulane University Law School, says that nothing less than letting the river go its own way will solve the land loss problem. “*Coast 2050* is history,” he says. “Katrina upped the ante so much. What has to be done now is to let the Mississippi River take its natural course and allow the full bed load of the river to rebuild the marsh.” He adds, “The problem with *Coast 2050* and other restoration plans is that they fail to halt wetland destruction in the same areas they are trying to restore. New canals, deeper canals, expanded ports are all on the table. No way that works.”

Indeed, if water control projects were destroyed and the Mississippi were allowed to take its natural course, it would inevitably become captured by the Atchafalaya River, which empties off the south-central coast of Louisiana. The combined flow and increased sediment load would help build up the most land-starved region of Louisiana’s coast. But if the Mississippi River were set free, one of today’s most important shipping channels would become water-starved from Baton Rouge to the gulf outlet.

So how would giant oceangoing ships reach the ports of South Louisiana? Houck recommends cutting an entirely new shipping channel from the gulf to the port complex of South Louisiana. Where would this channel be located? “That’s up to the engineers,” Houck says.

## A Muddy Future

No matter how it’s done, there is a new urgency to address the land loss problem. Senator Mary Landrieu (D–LA) has proposed a Hurricane Katrina Disaster Relief and Economic Recovery Act, cosponsored by Senator David Vitter (R–LA). This proposal would provide $250 billion for hurricane reconstruction, including $40 billion in ecosystem restoration and levee improvements. Some feel, though, that this proposal actually hurt Louisiana’s chances for restoration monies by appearing to reach for too much to fund a grab bag of projects. “Major restoration funding remains in doubt,” says Houck, “as indeed does the mega-question: how to restore.” At press time the bill had not made any progress.

It has taken a major hurricane to show the nation that it’s necessary to rebuild the wetlands and barrier islands of Louisiana. Although stakeholders have generally agreed on a plan to rehabilitate these resources, major funding has not been available. To restore New Orleans to health after Hurricane Katrina, though, it seems clear that the nation must find a way to fund the largest ecological rehabilitation project in U.S. history, a comprehensive effort to rebuild South Louisiana’s disappearing landscape.

## Figures and Tables

**Figure:**